# Experimental exposure to diesel exhaust increases arterial stiffness in man

**DOI:** 10.1186/1743-8977-6-7

**Published:** 2009-03-13

**Authors:** Magnus Lundbäck, Nicholas L Mills, Andrew Lucking, Stefan Barath, Ken Donaldson, David E Newby, Thomas Sandström, Anders Blomberg

**Affiliations:** 1 Department of Respiratory Medicine and Allergy, University Hospital, Umeå, Sweden; 2 Centre for Cardiovascular Science, Edinburgh University, Edinburgh, UK; 3 ELEGI Colt Laboratory, Centre for Inflammation Research, Edinburgh University, Edinburgh, UK

## Abstract

**Introduction:**

Exposure to air pollution is associated with increased cardiovascular morbidity, although the underlying mechanisms are unclear. Vascular dysfunction reduces arterial compliance and increases central arterial pressure and left ventricular after-load. We determined the effect of diesel exhaust exposure on arterial compliance using a validated non-invasive measure of arterial stiffness.

**Methods:**

In a double-blind randomized fashion, 12 healthy volunteers were exposed to diesel exhaust (approximately 350 μg/m^3^) or filtered air for one hour during moderate exercise. Arterial stiffness was measured using applanation tonometry at the radial artery for pulse wave analysis (PWA), as well as at the femoral and carotid arteries for pulse wave velocity (PWV). PWA was performed 10, 20 and 30 min, and carotid-femoral PWV 40 min, post-exposure. Augmentation pressure (AP), augmentation index (AIx) and time to wave reflection (Tr) were calculated.

**Results:**

Blood pressure, AP and AIx were generally low reflecting compliant arteries. In comparison to filtered air, diesel exhaust exposure induced an increase in AP of 2.5 mmHg (p = 0.02) and in AIx of 7.8% (p = 0.01), along with a 16 ms reduction in Tr (p = 0.03), 10 minutes post-exposure.

**Conclusion:**

Acute exposure to diesel exhaust is associated with an immediate and transient increase in arterial stiffness. This may, in part, explain the increased risk for cardiovascular disease associated with air pollution exposure. If our findings are confirmed in larger cohorts of susceptible populations, this simple non-invasive method of assessing arterial stiffness may become a useful technique in measuring the impact of real world exposures to combustion derived-air pollution.

## Background

The adverse health effects of air pollution have been studied intensely over the last 50 years. Epidemiological studies have identified a strong association between air pollution and increased cardiovascular and respiratory morbidity and mortality [1]. Airborne particles with a diameter of less than 2.5 μm (PM_2.5_) are considered the main cause of these adverse health effects [2]. In most countries, diesel exhaust (DE) is a major contributor to combustion-derived PM in urban areas [3], exposure to which appears to be an important trigger for acute myocardial infarction [4].

In recent years, there has been considerable interest and scientific research into the mechanisms that underpin these associations. We have recently demonstrated that exposure to DE causes exercise-induced ST-segment depression and impairs endogenous fibrinolytic function in patients with stable coronary heart disease [5]. Brook and colleagues suggest that exposure to air pollution may result in acute arterial vasoconstriction detected by ultrasonography of the brachial artery [6]. In complementary studies using venous occlusion plethysmography, we have reported impaired vasodilatation in response to intra-arterial infusion of endothelium-dependent and -independent agonists immediately after exposure to DE [7]. These sophisticated techniques are ideal for small scale experimental exposure studies. However, there is a need to develop a simple non invasive bedside test to assess the effects of real world exposures to ambient air pollution on vascular function in larger population studies.

The vascular endothelium plays a key role in the regulation of arterial tone. Traditional cardiovascular risk factors are associated with vascular dysfunction, reduced arterial compliance and increased central aortic pressure [8]. Enhanced large artery stiffness results in greater aortic systolic pressure, increased left ventricular afterload and reduced coronary blood flow [9]. Arterial stiffness may be the central pathological process in essential hypertension and is an independent predictor of mortality [10]. Applanation tonometry of the radial artery with pulse wave analysis is a well validated non invasive method of assessing arterial stiffness [11,12]. Smokers have been found to have stiffer arteries compared to non-smokers and develop an additional increase in arterial stiffness shortly after smoking one cigarette [13]. Given the similarity between cigarette smoke and combustion-derived air pollution, we hypothesised that exposure to DE would alter arterial tone and stiffness. The aim of this study was to assess the effects of exposure to diesel exhaust on arterial stiffness and blood pressure in healthy non-smoking volunteers.

## Methods

### Subjects

Twelve, non smoking, healthy men (mean age 26, range 21–30) participated in the study. All subjects had normal lung function, and reported no symptoms of respiratory tract infection for at least six weeks before or during the study. Exclusion criteria were regular medication, clinical evidence of atherosclerotic vascular disease, arrhythmias, diabetes mellitus, hypertension, renal or hepatic failure, asthma, occupational exposure to air pollution or an intercurrent illness. These subjects also participated in a separate study protocol that examined the effects of diesel exhaust exposure on thrombus formation and platelet activation; the results of that study are reported separately [14]. The study was approved by the local ethics committee, performed in accordance to the Declaration of Helsinki, and with the written informed consent of all participants.

### Study design

In a randomised double blind crossover study, subjects attended on two occasions at least one week apart and were exposed to either diesel exhaust or filtered air for 60 min. On each occasion, subjects underwent moderate exercise on a bicycle ergometer alternated with rest at 15 minutes intervals throughout, according to a previously described standard protocol [7]. The subjects abstained from alcohol for 24 hours and from food, tobacco and caffeine containing drinks for at least 4 hours before exposure. Immediately after exposure, arterial stiffness was performed with measures of pulse wave analysis (PWA) at 10, 20 and 30 minutes and pulse wave velocity (PWV) at 40 minutes after the end of exposure.

### Diesel-exhaust exposure

Temperature and humidity in the chamber were controlled at 22°C and 50% respectively.

The diesel exhaust was generated from an idling Volvo diesel engine (Volvo, TD45, 4.5 L, 4 Cylinders, 680 rpm) using Gasoil E10 (Preem, Sweden). After more than 90% of the exhaust was shunted away, the remainder was diluted with filtered air and fed into the exposure chamber at a steady-state concentration. Air in the chamber was continuously monitored for pollutants with exposures standardised using continuous measurement of oxides of nitrogen concentrations (NO_x_) to deliver a particulate concentration of approximately 350 μg/m^3^. Exposures were carried out in accordance to a well established protocol as previously reported. Workload during both exposures was adjusted to achieve the preset minute ventilation of 25 L/min/m^2^. We have previously ascertained by a questionnaire that there are no differences in reported symptoms (including those related to the upper airways) between the diesel and filtered air exposures and volunteers are unable to tell which exposure they have received. [15].

### Arterial Stiffness

Vascular studies were performed in a quiet, temperature controlled room with subjects resting in a supine position. Systolic and diastolic blood pressures were measured in duplicate using a semi automated non-invasive oscillometric sphygmomanometer, following a 10 min rest period and at 10, 20 and 30 minutes after each exposure.

Pulse wave analysis was performed using micromanometer applanation tonometry (Millar Instruments, Texas) of the radial artery at the wrist and the SphygmoCor™ system (AtCor Medical, Sydney) in accordance with the manufacturer's recommendations. Briefly, pulse wave analysis derives an aortic pulse pressure waveform from the radial artery wave via a mathematical transfer function. The arterial pressure waveform is a composite of the forward pressure wave created by ventricular contraction and a reflected wave generated by peripheral vascular resistance. The augmentation pressure (AP), is the difference between the second and first systolic peaks expressed in mm(Hg). The augmentation index (AIx), augmentation pressure as a percentage of the pulse pressure, is a measure of systemic arterial stiffness and wave reflection. The time to wave reflection (Tr) is reduced with increasing arterial stiffness, and provides a surrogate of aortic pulse wave velocity [16]. At least two independent waveform analyses were obtained from each subject, with measurements only accepted upon meeting SphygmoCor™ quality control criteria. Pulse wave velocity (PWV) was calculated by measuring the time for the pulse wave to travel between the carotid and femoral arteries. All measurements were performed by a single operator blinded to the nature of each exposure.

### Statistical analysis

The results were analysed using GraphPad Prism (GraphPad Software, USA) by 2-tailed Student's *t*-test and a repeated measure ANOVA where appropriate. Statistical significance was taken at P < 0.05. Data are presented as mean ± SEM.

## Results

Between DE exposures there were small variations in particle mass (330 ± 12 μg/m^3^), estimated particle number (1.26 ± 0.01 × 10^6^/cm^3^), oxides of nitrogen, NO_x _(2.78 ± 0.03 ppm), nitrogen dioxide (0.62 ± 0.01 ppm), nitric oxide (2.15 ± 0.03 ppm), carbon monoxide (3.08 ± 0.12 ppm) and total hydrocarbons (1.58 ± 0.16 μg/m^3^).

**Figure 1:**
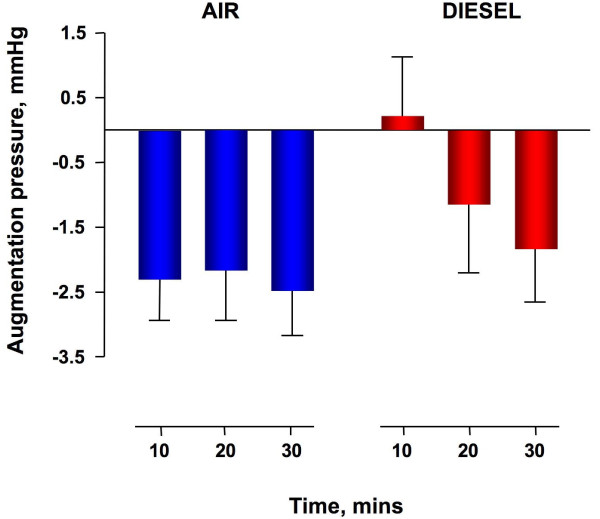
**Augmentation pressure increases immediately following exposure to dilute diesel exhaust as compared to filtered air and is normalised over 30 minutes (P = 0.01, diesel exhaust *versus *filtered air, repeated measures ANOVA, n = 12)**.

**Figure 2:**
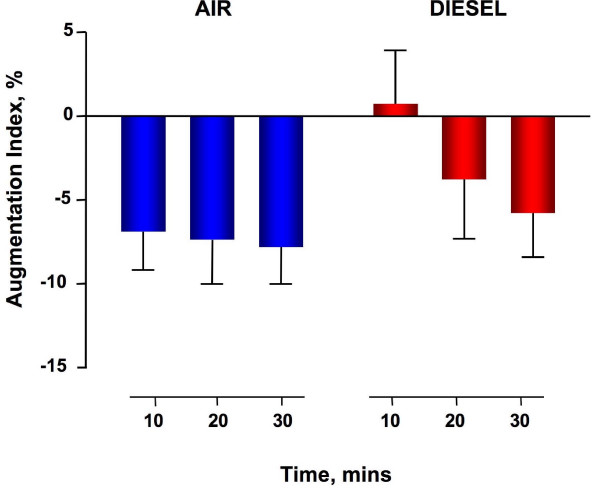
**Augmentation index increases immediately following exposure to dilute diesel exhaust as compared to filtered air and is normalised over 30 minutes (P = 0.02 respectively, diesel exhaust *versus *filtered air, repeated measures ANOVA, n = 12)**.

Diesel exhaust exposure did not affect heart rate or brachial diastolic and systolic blood pressure during exposure (data not shown) or throughout the study period (Table 1). Augmentation pressure (AP) and augmentation index (AIx) were generally low, reflecting compliant arteries in these young healthy volunteers [8]. AP and AIx increased immediately after diesel exhaust exposure compared to filtered air (p = 0.01 and p = 0.02 respectively) and normalised over 30 minutes (Figure 1 and figure 2). Time to wave reflection was reduced at 10 and 20 minutes (Table 1) consistent with an early increase in pulse wave velocity. However, formal measurement of carotid-femoral PWV 40 minutes following exposure was not different between exposures (5.1 ± 0.2 m/s versus 4.6 ± 0.1 m/s, P = ns).

**Table 1:** Effects of diesel exposure on heart rate, blood pressure and markers of arterial stiffness

	**Air** **(n = 12)**			**Diesel exhaust** **(n = 12)**			**Significance**
	
Time, mins	+10	+20	+30	+10	+20	+30	-
Heart rate, bpm	57 ± 2	55 ± 2	55 ± 2	57 ± 2	55 ± 2	54 ± 2	P = 0.66
Systolic BP, mmHg	118 ± 2	114 ± 2	115 ± 3	114 ± 2	114 ± 2	115 ± 3	P = 0.26
Diastolic BP, mmHg	67 ± 2	65 ± 2	66 ± 2	65 ± 2	68 ± 2	67 ± 3	P = 0.48
MAP, mmHg	80 ± 2	78 ± 2	79 ± 2	79 ± 2	81 ± 3	81 ± 3	P = 0.57
Augmentation pressure, mmHg	-2.3 ± 0.7	-2.2 ± 0.8	-2.5 ± 0.7	0.2 ± 0.9	-1.3 ± 0.9	-1.8 ± 0.8	P = 0.01
Augmentation index, %	-7.0 ± 2.3	-7.4 ± 2.6	-7.9 ± 2.2	0.8 ± 3.2	-3.8 ± 3.5	-5.8 ± 2.7	P = 0.02
Time to wave reflection, ms	187 ± 8	189 ± 11	180 ± 7	171 ± 11	163 ± 9	180 ± 8	P = 0.03

Values are Mean ± SEM;

diesel exhaust *versus *filtered air (repeated measures ANOVA)

BP = blood pressure; MAP = mean arterial pressure

## Discussion

Brief acute exposure to diesel exhaust is associated with a transient increase in arterial stiffness in healthy individuals. These changes in arterial stiffness occurred in the absence of any effect on peripheral arterial pressure. If our findings were confirmed in larger cohorts of susceptible populations, this simple non-invasive method of assessing arterial stiffness may become a useful technique in measuring the impact of real world exposures to combustion-derived air pollution.

As expected in our healthy young volunteers, blood pressure was in the normal range and there was no evidence of increased arterial stiffness following filtered air exposure. In contrast, exposure to diesel exhaust increased the augmentation pressure by 3 mmHg and augmentation index by 8%. Whilst these small changes are unlikely to be of significance in healthy individuals with normal cardiovascular function, these changes would be clinically important in susceptible individuals. Small increases in arterial stiffness can result in augmentation of central aortic systolic pressures that in turn increases left ventricular afterload and reduces diastolic coronary filling. In a patient with established hypertension or coronary artery disease, small changes in central aortic pressure may be sufficient to trigger an acute cardiovascular event. It is interesting to note that the effect of DE on augmentation index is of a similar magnitude to that of smoking a single cigarette [13].

To our knowledge, this is the first study to assess the effects of diesel exhaust air pollution on arterial stiffness. Our findings are consistent with those of Brook et al where a 2 hour exposure to concentrated ambient particles (CAPs) and ozone resulted in acute vasoconstriction in healthy volunteers [17]. It is entirely conceivable that this effect of CAPs on conduit vessel function would be associated with increased arterial stiffness and augmented central arterial pressures. Of note, Brook and colleagues found that exposure to CAPs caused a small but consistent increase in diastolic blood pressure by 2 mmHg [18]. Whilst changes in arterial stiffness in our cohort were not associated with an increase in blood pressure, this may be due to our shorter exposure, differences in particle composition between exposures, or the absence of any appreciable quantities of ozone in DE. Moreover, a recent field study revealed increased arterial stiffness following exposure to welding fumes [19]. Taken together, these studies underpin the findings in the present study.

A number of hypotheses have been presented to explain the mechanisms responsible for the adverse effects of PM on endothelial function and vasomotor tone [20]. Inhalation of PM causes oxidative stress in the airways and induces inflammatory response with upregulation of oxidative stress-sensitive pathways [20,21]. Oxidative stress triggers an acute inflammatory reaction locally in the airways with the potential to upregulate systemic inflammatory processes. Acute systemic inflammation has adverse effects on endothelial vasomotor function and increases arterial stiffness [21]. Whilst we did not measure oxidative stress or systemic inflammation in our current study, we have previously demonstrated that a similar DE exposure modifies airway antioxidants [15] and increases interleukin-6 and tumour necrosis factor-alpha concentrations in plasma [22]. Vascular tone is intimately regulated by the balance between reactive oxidant species and nitric oxide. Interestingly, there are reports in preclinical studies of nanoparticle translocation from the airways into the blood stream [23,24] with the possibility of a direct oxidative effect of PM on vascular function. Alternatively, oxidative stress may cause acute release of endothelin-1 from either pulmonary or systemic vascular endothelium [25]. Endothelin-1 is a potent vasoconstrictor that has been associated with both hypertension [26] and arterial stiffness [27] and further research into the impact of DE exposure on the endothelin system is anticipated.

Pulse wave analysis to measure arterial stiffness is a simple inexpensive and non-invasive bedside technique that allows repeated measures to determine the kinetics of air pollution-induced vascular dysfunction. Where plethysmography provides detailed characterisation of vascular mechanisms, it is time consuming, invasive, and cannot be used to assess acute or transient effects of exposure on vascular function. It is also not suitable for population-based studies.

Our studies to date have been based on carefully controlled exposures to diesel exhaust to avoid the potential for confounding that exists in observational studies. We believe this model is highly relevant both in the composition and magnitude of exposure for the assessment of short-term health effects in man. PM concentrations can regularly reach levels of 300 μg/m^3 ^and above in heavy traffic, occupational settings, and in the world's largest cities [28]. Exposure to 300 μg/m^3 ^for one hour increases a person's average exposure over a 24-hour period by only 12 μg/m^3^. Changes of this magnitude occur on a daily basis even in the least polluted cities, and are associated with increases in cardiorespiratory mortality [29].

Whilst we report transient changes in arterial stiffness as indicated by an increase in AP, AIx and a reduction in the time to wave reflection, we did not find any effect of exposure on PWV. This is perhaps not surprising as we measured PWV at 40 min; a time point where AP and AIx had returned to baseline values. Whilst the early reduction in time to wave reflection would suggest an immediate increase in pulse wave velocity, time to wave reflection is a derived parameter and only a surrogate marker of PWV. Carotid-femoral PWV is considered the gold-standard measurement of central arterial stiffness [30]. In subsequent studies, it will therefore be important to assess PWV immediately post exposure to validate our findings. Given the transient nature of the change detected by pulse wave analysis, it may be necessary to measure arterial stiffness and blood pressure during the exposure itself although this will undoubtedly present technical challenges. When exploring a novel mechanism it is important to first test the hypothesis in healthy volunteers to avoid any potential confounding by disease states and medications. However, as discussed, it is unlikely that small transient changes in arterial stiffness are of clinical relevance in our cohort. Therefore, subsequent studies would be of importance to address whether this effect of diesel exhaust on arterial stiffness is also applicable to women older people, and whether the effects would be even more pronounced in susceptible patient groups with pre-existing vascular dysfunction or hypertension.

## Conclusion

Brief exposure to diesel exhaust is associated with a transient increase in arterial stiffness in healthy individuals. This suggests that exposure to diesel exhaust impairs arterial function and provides insight into the association between air pollution and increased cardiovascular morbidity. If our findings are confirmed in larger cohorts of susceptible populations, this simple non-invasive method of assessing arterial stiffness may become a useful technique in measuring the impact of real world exposures to combustion-derived air pollution.

## Competing interests

The authors declare that they have no competing interests.

## Authors' contributions

AB, NM, KD, TS and DN coordinated and were responsible for the planning of the study. ML, AL and SB were responsible for the diesel exhaust exposures. ML carried out the arterial stiffness measurements. ML and NM analysed data and performed statistical analysis. The manuscript was written by ML, NM and AB and then read, corrected and approved by all authors.
